# Genome analysis identifies a spontaneous nonsense mutation in *ppsD* leading to attenuation of virulence in laboratory-manipulated *Mycobacterium tuberculosis*

**DOI:** 10.1186/s12864-019-5482-y

**Published:** 2019-02-12

**Authors:** Shyamasree De Majumdar, Kriti Sikri, Payel Ghosh, Neetika Jaisinghani, Malobi Nandi, Sheetal Gandotra, Shekhar Mande, Jaya Sivaswami Tyagi

**Affiliations:** 1 Department of Biotechnology, All India Institute of Medical Sciences, Ansari Nagar, New Delhi, 110029 India; 2 Bioinformatics Center, Savitribai Phule Pune University, Pune, Maharashtra India; 3 Cardiorespiratory Disease Biology Unit, CSIR-Institute of Genomics and Integrative Biology, New Delhi, India; 4 Amity Institute of Biotechnology, Amity University, Noida, Haryana India; 5 National Center for Cell Science, University of Pune Campus, Pune, Maharashtra India; 6 Centre for Bio-design and Diagnostics, Translational Health Science and Technology Institute, Faridabad, Haryana India; 7 Present affiliation: School of Biomedical Sciences, Ulster University, Coleraine, UK

**Keywords:** *M. tuberculosis*, Whole genome sequencing, Single nucleotide polymorphism, Phthiocerol dimycocerosates, *ppsD*

## Abstract

**Background:**

A previous laboratory study involving wild type, mutant and *devR*/*dosR* complemented strains of *Mycobacterium tuberculosis* reported the attenuation phenotype of complemented strain, Comp1. This phenotype was intriguing since the parental strain H37Rv, *devR* mutant (Mut1) and additional complemented strains, Comp9 and Comp11, were virulent in the guinea pig model.

**Results:**

Towards deciphering the mechanism underlying the attenuation of Comp1, a whole genome sequencing approach was undertaken. Eight Single Nucleotide Polymorphisms (SNPs) unique to the Comp1 strain were identified. Of these, 5 SNPs were non-synonymous and included a G➞A mutation resulting in a W1591Stop mutation in *ppsD* gene of the phthiocerol dimycocerosate (PDIM) biosynthetic cluster. Targeted sequence analysis confirmed this mutation in only Comp1 strain and not in wild type (H37Rv), *devR* knockout (Mut1) or other complemented (Comp9 and Comp11) bacteria. Differential expression of the PDIM locus in Comp1 bacteria was observed which was associated with a partial deficiency of PDIM, an increased sensitivity to detergent and a compromised ability to infect human THP-1 cells.

**Conclusions:**

It is proposed that a spontaneous mutation in the *ppsD* gene of Comp1 underlies down-modulation of the PDIM locus which is associated with defects in permeability and infectivity as well as virulence attenuation in guinea pigs. Our study demonstrates the value of whole genome sequencing for resolving unexplainable bacterial phenotypes and recommends the assessment of PDIM status while assessing virulence properties of laboratory-manipulated strains of *M. tuberculosis*.

**Electronic supplementary material:**

The online version of this article (10.1186/s12864-019-5482-y) contains supplementary material, which is available to authorized users.

## Background

*Mycobacterium tuberculosis* (*M. tb*) is characterized by the presence of a complex cell wall that is rich in high molecular weight lipids which are major determinants of virulence [1]. Phthiocerol dimycocerosates (PDIM), the most abundant of these lipids, are surface-exposed non-polar complex lipids that are made up of a mixture of long chain β-diols (phthiocerols) esterified by multimethyl-branched fatty acids named as mycocerosic acids [2–4]. PDIMs are produced by a limited group of mycobacterial species, most of which are pathogenic for humans such as *M. tb*, *M. bovis*, *M. leprae*, *M. ulcerans*, and *M. marinum* [2] and have attracted special attention due to the key role they play in the pathogenesis of tuberculosis and bacterial-host interactions [5–11]. A role for PDIM in tuberculosis (TB) pathogenesis was suggested over four decades ago when a PDIM deficient H37Rv-derived strain of *M. tb* was found to be attenuated in guinea pigs [10]. Furthermore, an avirulent strain of *M. tb* coated with a mixture of PDIM and cholesteryl oleate persisted longer than the uncoated strain in tissues of infected mice [12]. Sequencing of the *M. tb* genome revealed that a stretch of ~ 50 kb containing *ppsA-E, drrA-C, mas, mmpL7, fadD26, fadD28* and *papA5*, is dedicated to the biosynthesis and transport of PDIMs [13]. Genetic studies have established their role in virulence and pathogenesis. Mutants lacking or deficient in PDIM synthesis or translocation display altered colony morphology, increased membrane permeability phenotypes and are severely attenuated in animal models [8, 9, 14].

We have previously described the properties of *M. tb devR/dosR* complemented strains (Comp1, Comp9 and Comp11) that express different levels of DevR due to differences in promoter strength and gene copy number [15, 16]. The *devR* mutant strain (Mut1) expresses only the DevR N-terminal signal receiver domain and lacks the DNA binding domain [17]. Mut1 bacteria were defective in expression of the DevR regulon and survival under hypoxia. Among the Comp strains, while Comp9 rescued the hypoxia adaptation defect, the Comp1 and Comp11 strains were unable to do so as efficiently. Intriguingly, all of these strains (H37Rv, Mut1, Comp9 and Comp11), with the exception of Comp1 strain, were virulent in guinea pigs whereas Comp1 was highly attenuated [16].

The present study aimed to decipher the mechanism underlying the puzzling attenuation phenotype of Comp1. Whole genome sequencing of Comp1 revealed the presence of 8 Single Nucleotide Polymorphisms (SNPs) unique to the Comp1 strain. Of these, 5 SNPs were non-synonymous and included a spontaneous nonsense mutation, W1591Stop, in the *ppsD* gene of the PDIM biosynthetic cluster. Transcriptome analysis revealed differential expression of the PDIM locus in Comp1 bacteria that was associated with a partial deficiency of PDIM, an increased sensitivity to an anionic detergent (sodium dodecyl sulfate) and a compromised ability to infect THP-1 cells. We propose that down-modulation of the PDIM locus in Comp1 bacteria due to a spontaneous mutation in the *ppsD* gene is the likely molecular mechanism underlying attenuation and associated defects in Comp1 bacteria. Based on the spontaneous generation of PDIM deficient bacteria during in vitro culture, we recommend a routine assessment of PDIM while characterizing virulence of wild type and laboratory-manipulated *M. tb* strains.

## Methods

### Characteristics of *M. tb* strains and culture conditions

The *M. tb* strains used in this study are described in Table 1. Frozen bacterial stocks were sub-cultured twice until logarithmic phase (A_595_ ~ 0.4) in Dubos medium containing 0.5% BSA, 0.75% Dextrose, 0.085% NaCl and 0.1% Tween 80 (DTA medium).

**Table 1:** Virulence characteristics of strains used in the study^a^

Strain	Background	DevR expression	Body weight score	Organ weight ratio		Organ visual score		Bacterial load		Virulence index
				Spleen	Lung	Spleen	Lung	Spleen	Lung	
H37Rv (wild type)	–	+	1.000	1.000	1.000	1.000	1.000	1.000	1.000	1.000
Mut 1	H37Rv disrupted in *devR* by Kan^R^-cassette insertion	–	1.004	0.827	1.048	0.903	0.938	0.269***	2.687	1.096
Comp1	Mut1	+	1.251*	0.357***	0.610***	0.069***	0.219*	0.00002***	0.0003*	0.358
Comp9	Mut1	+	1.019	0.785	1.155	0.787	0.521	0.222***	1.165	0.808
Comp11	Mut1	+	0.980	0.851	0.975	0.972	0.938	0.227***	2.341	1.041

^a^The data is compiled from our earlier publication [16]. A score of 1 is assigned to all parameters (body weight score etc.) and to the overall virulence index at 13 weeks that were measured during guinea pig infection with H37Rv. The relative scores for other strains are calculated with reference to that of H37Rv. [*** *p*value < 0.001; * *p*value < 0.05]

### Whole genome sequencing (WGS) using Illumina HiSeq-2000 platform

TrueSeq DNA Sample Preparation Kit (Illumina) was used to prepare the WGS library from purified genomic DNA sample of Comp1 strain. The quality and concentration of isolated DNA was assessed for OD_260_/OD_280_ ratio > 1.8, OD_260_/OD_230_ ratio > 1.9, DNA concentration between 250 to 500 ng/μl and no visible evidence of DNA degradation or contamination with RNA. Approximately 1.5 μg of high quality genomic DNA was used to generate fragments of size 300–400 bp by Covaris. Fragments were end-repaired by mixing with End Repair Mix and purified by Ampure XP reagent (Beckman Coulter). These fragments were adenylated and ligated to DNA Adapter Indexes for multiplexing with DNA Ligase Mix. The ligation products were purified and were subsequently enriched by PCR amplification with PCR Master Mix (TrueSeq DNA Sample Prep Kit, Illumina) according to manufacturer’s recommended protocol. The quality and quantity of the genomic DNA library thus obtained was assessed by 2100 Bioanalyzer (Agilent) and real time PCR with Kapa Library Quant Kit (Kapa Biosystems) in ABI 7900HT system (Life Technologies). Genomic DNA library of fragment size between 400 to 500 bp was selected and sequenced on the HiSeq-2000 System (Illumina) using TrueSeq PE Cluster Kit v3 and TrueSeq SBS Kit v3 (Illumina).

### Mapping and analysis of WGS data of Comp1 strain

The sequenced reads were mapped against the genome of *M. tb* H37Rv (NCBI accession number NC_000962) using three different mapping programs, Bowtie2 [18], Burrows-Wheeler aligner (BWA) [19] and MIRA [20]. The mapping results obtained with the Bowtie2 program, after removal of low-quality reads (Q < 20), represented an average genome coverage of 820x. A subset of the total reads (~ 25%) were mapped to the reference genome using BWA (version 0.6.2), and MIRA (version 3.4), to check whether any gaps had been created in the mapped reconstruction of the Comp1 genome due to differences in the mapping algorithms. MIRA was used for performing both reference-based mapping and de novo assembly. The contigs generated through de novo assembly were further aligned to the mapping-based assembly in order to cover gaps in the reference-based mapping. It may be noted that most of the gaps remaining after this exercise were present only within multicopy regions, such as insertion sequences (IS), transposons, repeat-containing proteins (Rv1587c, Rv1588c), etc.

Single nucleotide polymorphism (SNP) calls and consensus sequence generation were subsequently performed using the pileup function in SAMtools (version 0.1.16) [21] with the mapping outputs of Bowtie2 and BWA. The variant-calling program integrated in the MIRA software was used to list all the SNPs/ InDels (Insertion and deletion of bases) obtained from the mapping results. The whole genome sequencing data of Comp1 has been submitted to the NCBI Sequence Read Archive (SRA; http://www.ncbi.nlm.nih.gov/sra/) under accession number PRJNA492224.

### Polymerase chain reaction and sanger sequencing

DNA isolated from *M. tb* strains H37Rv, Mut1, Comp1, Comp9 and Comp11 were subjected to amplification using the primer pair *ppsD*seqF and *ppsD*seqR (Additional file 1: Table S1). The template was denatured at 90 °C for 30 s, followed by primer annealing at 65 °C for 30 s and elongation at 72 °C for 30 s. The amplified product was sequenced by Sanger methodology using *ppsD*seqF primer (Additional file 1: Table S1).

### RNA extraction and quantitation of bacterial gene expression

*M. tb* strains were cultured as described above with shaking at 220 rpm till A_595_ ~ 0.2–0.3. A 50 ml aliquot was centrifuged and RNA was isolated using TRI reagent method as described [16]. Reverse transcription was performed on the total extracted RNA with random hexamer primers and cDNA High capacity Reverse Transcriptase kit (ABI, USA). mRNAs were quantitated by real-time PCR using gene-specific primers (Additional file 1: Table S1) in MyIQ thermal cycler (Biorad, USA). For whole genome transcriptome, the data deposited with NCBI GEO datasets (Accession number GSE30264) was analysed as described previously [16].

### Detection of apolar lipids

*M. tb* log phase cultures (5 ml) were labelled for lipids by pulsing them with 0.5 μCi of Propionic acid [1-^14^C] sodium salt (specific activity 54 mCi/ mmol; American Radiolabeled Chemical, Inc) or 0.5 μCi of Acetic acid [1-^14^C] sodium salt (specific activity 31.7 mCi/ mmol; Board of Radiation and Isotope Technology, Department of Atomic Energy, Government of India). Cultures were harvested after 16 h of radioactive pulse by centrifugation and the pellets were heat inactivated at 95 °C for 30 min before proceeding for lipid extraction. Apolar lipid extract was prepared by adding 2 ml of methanolic solution of 0.3% sodium chloride and 1 ml of petroleum ether (60–80 °C) to the cell pellet. Extraction was performed by mixing in a tube rotator at room temperature for 30 min followed by centrifugation at 2500 rpm for 10 min. After phase separation, the upper layer consisting of apolar lipids was carefully removed and collected in a separate vial. One ml of petroleum ether was added to the remnant lower layer, vortexed and mixed for another 15 min. Phase separation was performed again by centrifugation and the upper layer obtained this time was pooled with the previous one. The upper layer comprising of apolar lipids was dried at 60 °C and resuspended in chloroform: methanol (2:1, *v*/v). Total radioactivity was measured as counts per minute (cpm) in a scintillation counter. Samples were dried and re-dissolved in chloroform: methanol (2:1, v/v) to achieve equal cpm/ml of lipid extract. Approximately, 10,000 counts from all the extracts belonging to the different cultures were spotted on thin layer chromatography (TLC) plate (silica gel 60) and developed uni-dimensionally in the solvent system petroleum ether: diethyl ether (90:10). The lipids were visualized with a Typhoon FLA 700 Phosphorimager.

### In vitro assessment of viability of *M. tb* strains and SDS susceptibility assay

*M. tb* cultures were grown to mid-logarithmic phase (A_595_ ~ 0.4) in DTA medium. The culture cell densities were adjusted to an A_595_ of 0.005 (in 10 ml cultures). Sodium dodecyl sulfate (SDS) was added at a final concentration of 0.05 and 0.1% and the cultures were incubated for 10 days. Aliquots were collected after 0, 1, 4 and 10 days and the number of viable bacteria was estimated by plating on 7H11 agar medium supplemented with OADC. Total CFUs were finalized after 6 weeks.

### THP-1 infection assay

The inocula for infection were prepared by culturing *M. tb* strains with shaking to A_595_ ∼ 0.6 in DTA medium. THP-1 cell line was maintained in RPMI 1640 medium supplemented with 10% fetal calf serum. Briefly, THP-1 cells were seeded at 0.25 × 10^6^ cells per well in 24-well tissue culture plates and were differentiated by the addition of phorbol 12-myristate acetate (30 nM) for 24 h. The monolayers were infected with *M. tb* strains at a low multiplicity of infection (1 bacterium per 50 macrophages) for 20 h and washed with incomplete RPMI 1640. Fresh complete RPMI 1640 was added to each well and the plates were incubated at 37 °C for upto 7 days. Intracellular viable bacteria on day 0, 1, 4 and 7 post-infection were quantified by lysis of the monolayers with 0.025% SDS, and plating as described above. Infectivity is expressed as a percentage of bacteria internalized on day 1 compared to the number of bacilli used for infection.

### Statistical analysis

Statistical analysis wherever applicable was performed using Student’s t-test unless otherwise mentioned.

## Results

### *M. tb* Comp1 strain is attenuated in guinea pigs

We previously reported that *devR* complemented strain Comp1, was severely attenuated in guinea pigs, whereas the parental H37Rv and the *devR* mutant (Mut1) strains were virulent [16]. Two other complemented strains, Comp9 and Comp11, were not attenuated in guinea pigs in the same study [16]. An assessment of this previously generated virulence data [16] indicated that Comp1 strain was attenuated. The attenuation of Comp1 could not be explained because the parental H37Rv and Mut1 strains exhibited a virulent phenotype (Table 1, described originally by Majumdar et al. [16]).

### Whole genome sequencing of Comp1 strain reveals a nonsense mutation in *ppsD*

**Fig. 1:**
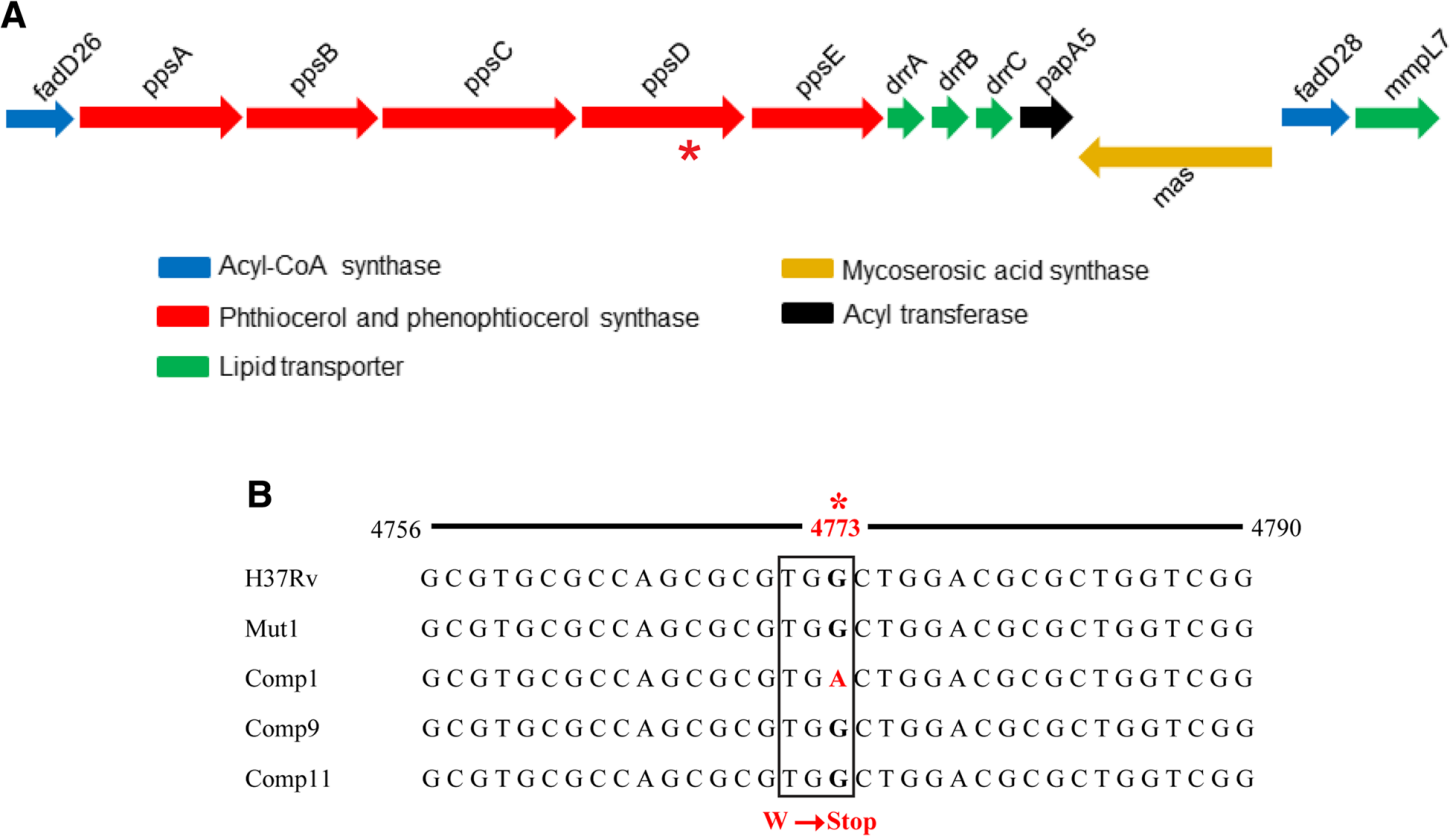
Analysis of *ppsD* sequence in *M. tb* strains. **a** Schematic representation of the PDIM locus of *M. tb* H37Rv. **b** Confirmation of SNP in *ppsD* gene of Comp1 by PCR amplification and sequencing. *indicates the position 4773 of G➞A SNP resulting in a TGA nonsense codon at W1591 (boxed)

Towards deciphering the underlying basis for the attenuation of Comp1, its genome was sequenced. The SNPs in Comp1 obtained by mapping the sequenced reads to *M. tb* H37Rv genome (NCBI ID: NC_000962) were compared against the reported SNPs [22]. A total of 171 probable SNPs and 21 InDels in Comp1 were detected (Additional file 2: Table S2 and Additional file 3: Table S3) and compared with the ‘erroneous’ sites identified previously [22]. Of these, 57 SNPs and 15 InDels were present in the ‘erroneous’ variations list and not analyzed further. Among the remaining 114 SNPs, 106 SNPs, as well as all the 6 remaining InDels were previously reported for other *M. tb* strains (GMVT Database [23]). Based on this comparative analysis with previously reported genome sequences [22, 23], 8 SNPs that were not reported for any *M. tb* strain, were assumed to be unique to the Comp1 strain (Table 2). Among these 8 SNPs, 5 variants were non-synonymous, which occur in genes corresponding to an oxidoreductase, a polyketide synthase gene *ppsD*, a gene involved in acetylation, a PE-PGRS57 gene (function unknown) and a conserved hypothetical gene (Table 2). The SNP occurring in the *ppsD* gene lies within the PDIM locus (Fig. 1a). PpsD is a modular polyketide synthase protein and along with PpsA-E is responsible for the synthesis of the PDIM lipid core. PpsD is an 1827 amino acids-long protein, and the SNP in Comp1 occurs at codon 1591 (TGG➞TGA/ W1591➞STOP), and would result in a truncated protein. Furthermore, a premature nonsense codon in *ppsD* is expected to result in transcriptional polarity and reduced expression of the downstream genes of the *fadD26-papA5* operon in Comp1 bacteria.

**Table 2:** SNPs unique to *M. tuberculosis* Comp1 strain^a^

SNP location	Rv number	Gene Name	Base change	Amino Acid Change	Gene Description	Protein Function	Type (N/S)^b^
161047	*Rv0133*		G/A	G60D	Acetyltransferase	Acetylation, substrate unknown	N
608331	*Rv0516c*		A/C	M69 L	Conserved hypothetical protein	May be involved in regulating sigma factor	N
635633	*Rv0543c*		C/T	A81A	Conserved hypothetical protein	Function unknown	S
958922	*Rv0861c*	*ercc3*	C/A	A410A	DNA helicase 3	Involved in nucleotide excision repair	S
1711627	*Rv1520*		C/T	Y200Y	Conserved hypothetical protein	Function unknown	S
2006032	*Rv1771*		A/G	Q291R	Oxidoreductase	Possibly involved in biosynthesis of L-ascorbic acid (vitamin C). Oxidizes L-gulono-1,4-lactone.	N
3267020	*Rv2934*	*ppsD*	G/A	W1591Stop	Phenolpthiocerol synthesis type-I polyketide synthase	Involved in phenolphthiocerol and phthiocerol dimycocerosate (dim) biosynthesis	N
3948356	*Rv3514*	PE_PGRS57	A/G	S855G	PE-PGRS family protein PE_PGRS57	Function unknown	N

^a^The SNPs unique to Comp1 were identified by comparing the whole genome sequences of Comp1 vs. H37Rv strain

^b^*N* Non-synonymous mutation, *S* Synonymous mutation

The genomic region encompassing the *ppsD* mutation in Comp1 was amplified by PCR from DNA of all the strains and analyzed by Sanger sequencing. The nonsense codon (TGA) was uniquely detected only in Comp1 DNA while the other strains, namely H37Rv, Mut1, Comp9 and Comp11, contained wild type *ppsD* gene sequence (Fig. 1b).

### PDIM biosynthesis cluster is differentially expressed in *M. tb* Comp1

**Fig. 2:**
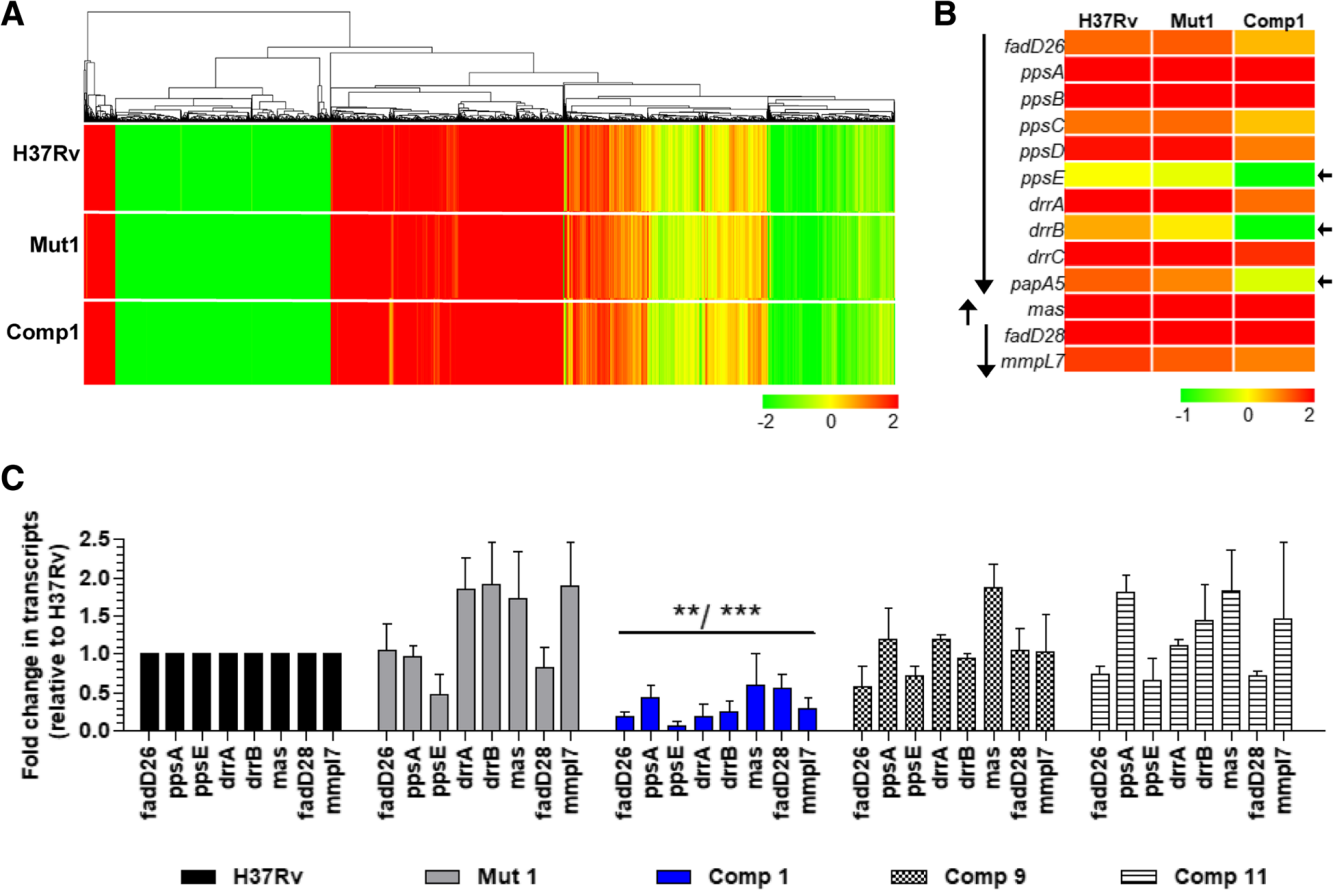
Differential expression of PDIM cluster genes in *M. tb* Comp1. **a** Hierarchical clustering of whole transcriptomes of *M. tb* H37Rv, Mut1 and Comp1. (Expression values are shown as mean fold change from 3 replicates normalized with respect to *M. tb* genomic DNA; GEO dataset Accession number GSE30264). **b** Heat map depicting expression values of genes belonging to the PDIM cluster, extracted from the microarray data shown in panel A. The vertical arrows on the left depict the arrangement of operons in the PDIM biosynthesis cluster [8]. The horizontal arrows on the right depict genes that were expressed at a lower level as compared to H37Rv. **c** RT-qPCR analysis of selected PDIM genes in *M. tb* strains. Relative transcript levels were calculated from C_t_ values normalized for 16S rRNA expression and then expressed with respect to that of H37Rv. Mean transcript level ± SD determined from three independent cultures is shown. [**, *p*value < 0.01 and ***, *p*value < 0.001 in comparison to H37Rv]

We next analyzed the expression of PDIM cluster to decipher the effect of *ppsD* nonsense mutation in Comp1 on expression of the *pps* locus. The previously published aerobic transcriptome profiles of H37Rv, Mut1 and Comp1 strains (GEO Accession number GSE30264) were found to be broadly similar (Fig. 2a). A decreased expression of several genes belonging to the PDIM cluster in Comp1 was observed (Fig. 2b). Reverse transcriptase-qPCR validation of mRNA confirmed the transcription defect; overall, transcript levels of the PDIM synthesis and transport genes were repressed 3 to 5-fold in the Comp1 strain in comparison to all other strains. Significant perturbation in gene expression was observed in *fadD26-papA5* operon which includes the *ppsD* gene (*p*value ranging between < 0.01 and < 0.001) and not in divergently transcribed *mas* gene sequence and *fadD28-mmpl7* operon (Fig. 2b and c). A higher expression of PDIM cluster genes has been previously correlated with the virulence of *M. tb* strains [8, 9]. Thus, the identification of a *ppsD* mutation in Comp1 was consistent with lowered expression of PDIM locus and attenuation of this strain.

### Comp1 strain is deficient in PDIM

**Fig. 3:**
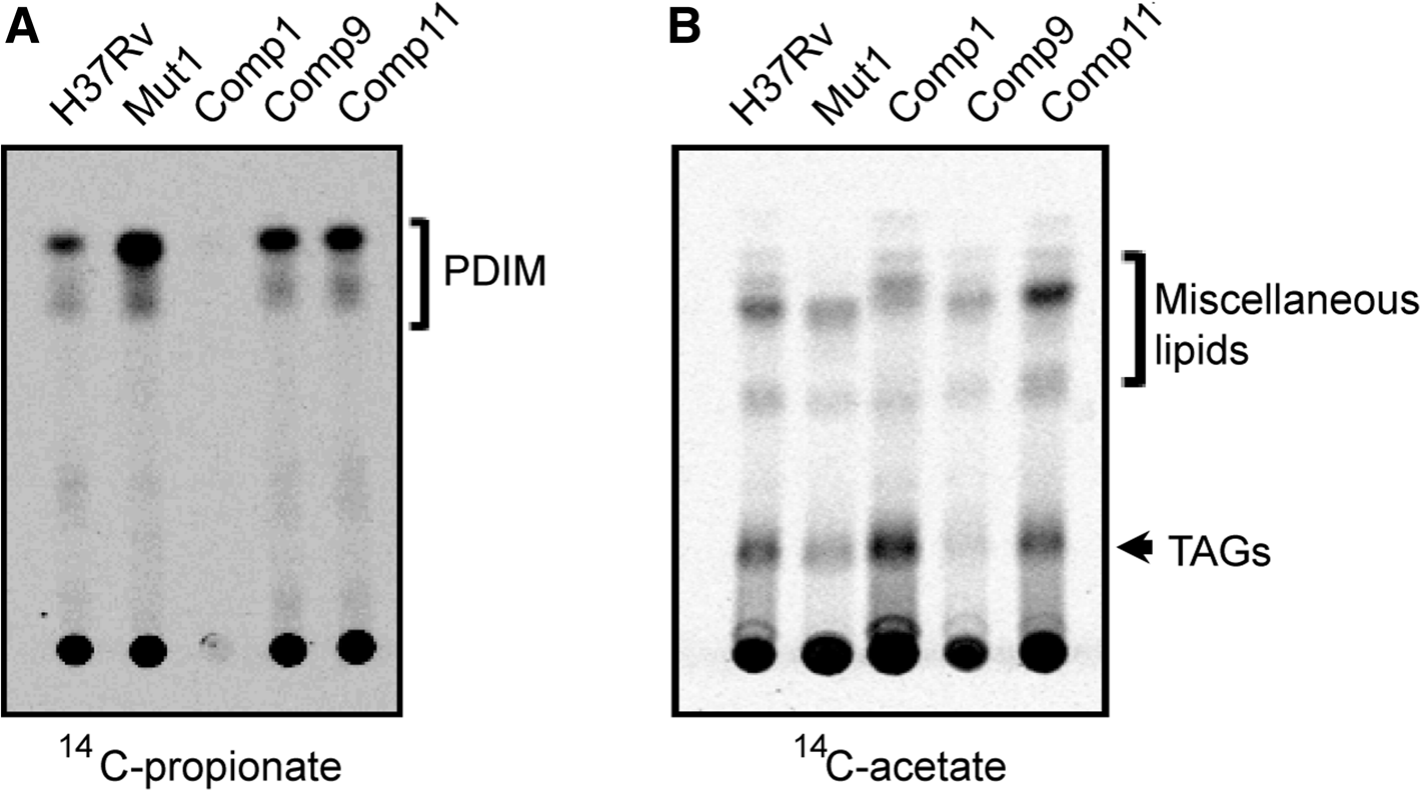
Analysis of apolar lipids by radioactive labeling. **a** Apolar lipids of *M. tb* strains analyzed by ^14^C-propionate labeling. **b** Total lipids of *M. tb* strains analyzed by labelling with ^14^C-actetate. Equal counts were loaded onto TLC and detected by phosphorimaging except in case of ^14^C-propionate labelled lipids of Comp1 where labelling was defective

To determine whether the lowered transcription of the PDIM cluster resulted in altered PDIM production, nonpolar surface lipids that incorporated ^14^C-propionate or ^14^C-acetate were extracted from *M. tb* strains and analyzed by TLC. ^14^C-propionate labelling is commonly used to assess the synthesis of branched lipids including PDIMs in *M. tb* [24]. Comp1 exhibited an ~ 115-fold defect in the incorporation of ^14^C-propionate with respect to *M. tb* H37Rv (*p*value < 0.001; Fig. 3a and Additional file 4: Table S4), which pointed to a possible defect in PDIM synthesis. Next, the cultures were labelled with ^14^C-acetate to rule out a generalized defect in lipid synthesis; no significant difference was observed in incorporation of acetate label in Comp1 and other strains (Fig. 3b). These findings indicate that Comp1 has a severe and specific defect in the synthesis of PDIM virulence lipids.

### PDIM deficiency increases cell wall permeability in Comp1 bacteria

**Fig. 4:**
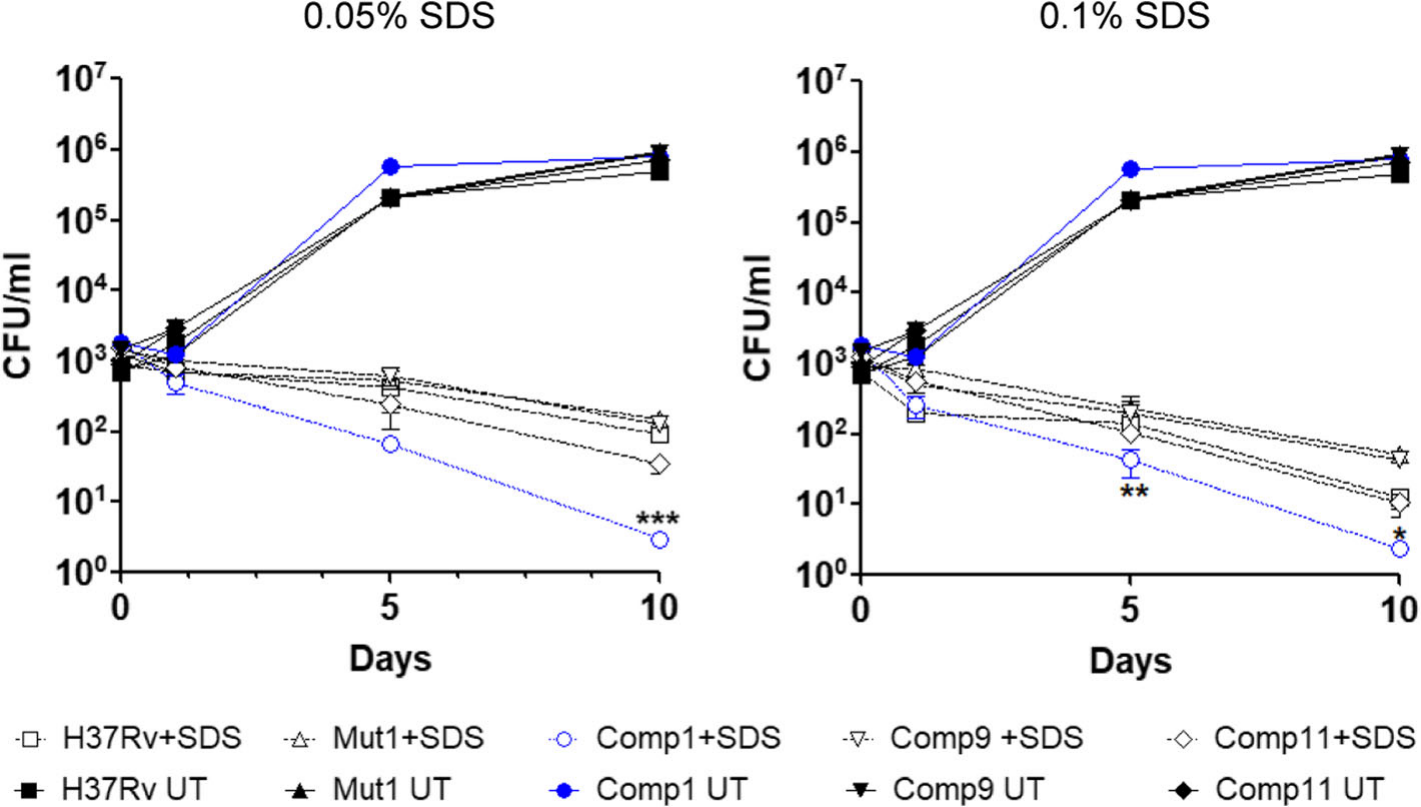
In vitro susceptibility of *M. tb* strains to SDS. Viability of various *M. tb* strains in the presence of SDS. The solid lines represent the untreated cultures and the dotted lines represent SDS-treated cultures. [*, *p*value < 0.05, **, *p*value < 0.01 and ***, *p*value < 0.001 in comparison to H37Rv]

PDIM-less mutants were earlier shown to be more sensitive to SDS than the wild type strain. This phenotype is consistent with alterations in the cell wall structure and an increase in cell wall permeability [8]. In order to determine whether the PDIM synthesis defect in Comp1 bacteria was associated with a functional defect, we assessed the permeability of Comp1 bacteria exposed to SDS. While a rapid decrease in the number of viable bacteria was observed for all the strains, Comp1 bacteria treated with SDS (0.05 and 0.1%) were significantly more sensitive to detergent (Fig. 4; *p*value < 0.05). These findings demonstrated that PDIM deficiency was associated with an increased cell wall permeability in Comp1 bacteria.

### PDIM deficiency compromises the infectivity of Comp1 strain

**Fig. 5:**
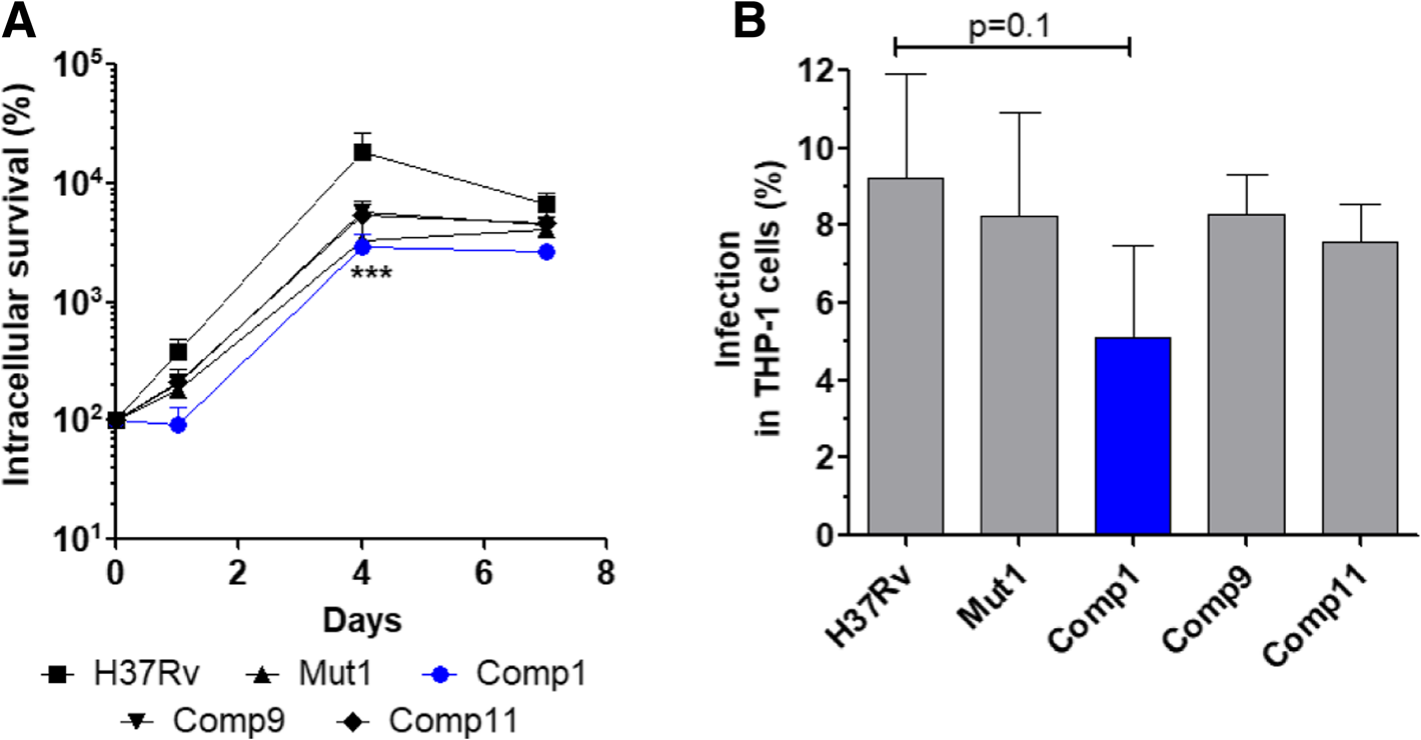
Intracellular survival and infectivity of *M. tb* strains. **a** Intracellular survival of *M. tb* strains in THP-1 infection model. Intracellular bacteria were recovered from THP-1 cells by lysis at various time points and bacterial survival was determined by CFU analysis. **b** Infectivity of *M. tb* strains in THP-1 cells analyzed after infection. Results are shown as the mean ± SD of 3 independent experiments. [***, *p*value < 0.001 in comparison to H37Rv]

PDIM was reported to participate in the process of macrophage infection at the initial stage of pathogen entry into cells [25]. Therefore, we assessed the infectivity and intracellular survival of various *M. tb* strains in the THP-1 cell infection model. Comp1 exhibited a defect in intracellular survival in comparison to H37Rv (*p*value < 0.001; Fig. 5a). Moreover, Comp1 bacteria displayed a reduced albeit non-significant ability to infect THP-1 cells in comparison to H37Rv, Mut1 and other Comp strains (Fig. 5b, *p*value = 0.1). Taking together these findings and previously published observations [25], the attenuation of Comp1 in guinea pigs could be ascribed, at least in part, to compromised infectivity of PDIM deficient bacteria.

## Discussion

Previous characterization of a panel of *devR* complemented (Comp) strains of *M. tb* revealed that Comp1, but not H37Rv, Mut1 or any other Comp strains, was attenuated in guinea pigs [16]. The present study aimed to utilize a functional genomics approach to explain this puzzling phenotype of Comp1 bacteria. Sequencing of the Comp1 genome revealed a nonsense mutation at codon 1591 that altered a tryptophan codon to a stop codon in *ppsD*, a component of PpsA-PpsE type 1 polyketide synthase which synthesizes the phthiocerol backbone of PDIM. Interestingly, the position of this SNP is otherwise a conserved one (occupied mostly by aromatic amino acids), and is part of a keto-acyl reductase domain (SMART domain ID: PKS_KR) that modifies the β-keto-thioester intermediate during PDIM synthesis [26]. Gene expression analysis further revealed a lower level of transcripts from the PDIM cluster in Comp1 compared to the other strains. The ~ 50 kb gene cluster involved in PDIM biosynthesis and transport is organized into three transcriptional units: (i) a major unit spanning ~ 32 kb from *fadD26* to *papA5 genes* (*Rv2930-Rv2939*) and including *ppsA-E*, (ii) a second unit containing only the *mas* gene from the complementary strand and, (iii) a third unit including the *fadD28* and *mmpL7* ORF [8, 9]. The nonsense mutation in *ppsD* was associated with lowered transcription of the downstream genes (*ppsE* and *drr*) likely due to transcriptional polarity effects. The selective deficiency in PDIM synthesis was indicated by a defect in ^14^C-propionate labeling of branched lipids. Taking together the results of genome sequencing, expression data and PDIM analysis, the attenuation of Comp1 is attributed to this *ppsD* mutation and deficiency in PDIM arising out of this spontaneous mutation.

A link between PDIM down-modulation and its function was established when Comp1 exhibited an increased susceptibility to SDS and decreased infectivity in THP-1 cells. The mycobacterial cell wall is characterized by its unusually low permeability to antibiotics, drugs and detergents which is attributed to cell wall lipids including PDIMs [8, 27]. The increased sensitivity of Comp1 to detergent indicates that cell wall permeability was altered and is consistent with the lowered levels of PDIM in this strain. Several roles have been ascribed to PDIM in *M. tb* virulence, such as involvement in bacterial phagocytosis, preventing phagosomal acidification, conferring protection against reactive nitrogen intermediates and modulating the early immune response to infection [28]. Comp1 bacteria exhibited lower infectivity compared to other strains in the THP-1 cell infection model and this defect may have contributed to its attenuation phenotype. Comp1 bacteria do not exhibit a growth defect during intracellular residence, thereby ruling out growth kinetics as a factor contributing to its attenuation. However, lower infectivity of THP-1 cells suggests a scenario that can explain the attenuation phenotype observed in guinea pigs [16] wherein Comp1 bacteria could be gradually cleared over multiple cycles of infection and result in overall reduced bacterial load. Our previous [16] and present findings are consistent with published reports of virulence attenuation wherein PDIM mutants of *M. tb* and *M. bovis* that were attenuated in both lungs and spleen of mice [14, 29–31].

It is not evident how Comp1 strain acquired a mutation in the PDIM locus. One possibility is that the *ppsD* mutation arose spontaneously during in vitro culture of Comp1 and its attenuation phenotype was independent of DevR. BLASTN analysis of the *ppsD* locus containing the G➞A SNP at position 4773 of the gene indicated that this nucleotide change was not found in any of the other strains of *M. tb* complex (taxonomy id: 77643), ruling out an evolutionary basis for this mutation (data not shown) and thereby established its random appearance in the genome of Comp1 strain. The virulence attenuation phenotype of Comp1 was not reversed by animal passage [15], suggesting that the acquired mutation is random and stable. Extended in vitro culturing of *M. tb* is reportedly associated with a loss in the ability to synthesize PDIM and likely to generate a heterogeneous population of PDIM-positive and -negative bacteria [29, 32]. There are reports in literature of spontaneously arising mutations and deletions in the PDIM biosynthetic pathway, including *pps* genes, that are associated with PDIM deficiency [9, 29, 33]. Furthermore, numerous studies have demonstrated a direct link between the loss of PDIM and attenuation of virulence in animal models [9, 29].

The other 4 gene loci in Comp1 containing non-synonymous SNPs encode an acetyltransferase (Rv0133), a conserved hypothetical gene (Rv0516c), an oxidoreductase (Rv1771) and a PE_PGRS family protein (Rv3514). To the best of our knowledge, there are no reports in the literature of their association with a virulence phenotype. Although the observed virulence attenuation phenotype of Comp1 can be explained by the *ppsD* mutation and its downstream effects on PDIM production, we do not exclude the possibility that other aspects of bacterial physiology and virulence might have been affected by one or more of the other four mutations detected in Comp1 by genome sequencing. The association of a randomly generated mutation in the PDIM gene cluster of Comp1 bacteria with loss of virulence points to the importance of assessing the PDIM status of *M. tb* strains while characterizing their virulence properties, especially when the outcomes of genetic analysis are not readily explainable.

## Conclusions

A functional genomics approach was successfully utilized to identify a spontaneous mutation in *ppsD* gene belonging to the PDIM cluster which led to down-regulation of PDIM synthesis in *M. tb* Comp1 bacteria and associated defects in permeability and infectivity. These findings suggest the defect in PDIM levels as a plausible molecular mechanism underlying Comp1 virulence attenuation in guinea pigs.

## Additional files

Additional file 1: **Table S1.** Primers used for qPCR assay and sequencing. (DOCX 15 kb)

Additional file 2: **Table S2.** List of SNPs identified *M.tb* Comp1 strain with respect to *M.tb* H37Rv. (DOCX 42 kb)

Additional file 3: **Table S3.** Insertions and Deletions (InDels) identified in *M. tb* Comp1 strain with respect to *M. tb* H37Rv. (DOCX 20 kb)

Additional file 4: **Table S4.** Total counts per minute (cpm) obtained on incorporation of ^14^C-propionate into *M. tb* cultures. (DOCX 14 kb)
