# Supplementary material for: Genome analysis identifies a spontaneous nonsense mutation in ppsD leading to attenuation of virulence in laboratory-manipulated Mycobacterium tuberculosis
Source: BMC Genomics. 2019 Feb 12;20:129. doi: 10.1186/s12864-019-5482-y (PMC6373159; doi:10.1186/s12864-019-5482-y)
Supplement: Supplementary file 1 — Table S1. Primers used for qPCR assay and sequencing. (DOCX 15 kb) [file 12864_2019_5482_MOESM1_ESM.docx]

**Table S1: Primers used for qPCR assay and sequencing**

| **Primers** | **Sequence (5’ 🡪 3’)** | **Assay** |
| --- | --- | --- |
| *ppsD* seq F  *ppsD* seq R | GCT GAC TGC GAG TCG ACT GGT GG  GCG ACG CCT ACC TCC GAC CAC G | Sanger sequencing |
| *ppsA* F  *ppsA* R | CAC ACG ATC GAC TGG CAA CC  GAT TAT CGC GAC GGG TCC TG | qPCR |
| *ppsE* F  *ppsE* R | AGA GTC GCA GAC CGA GGT TA  GCT GAT CGC CAT CAA AGA AT | qPCR |
| *drrA* F  *drrA* R | TGG TCG TCT GTG GGG ACT GA  GCC TCT TTC CGG CAT GTA CG | qPCR |
| *drrB* F  *drrB* R | CGT CGC CAG CAA CTT AGG GCA  ATT GAC GCC TAG CAG CGA AT | qPCR |
| *fadD26* F  *fadD26* R | GAT GAG CCC AAT GTC ATT  CCA GCT CGA AGG CGA AAT | qPCR |
| *fadD28* F  *fadD28* R | AAC TCG GCA ACA TAC TGA CC  CCT CTC CTG CAG ATT GAA GC | qPCR |
| *mmpL7* F  *mmpL7* R | GTC GGA ATA CCT GGA AAC GA  ACC ACG ATG TCG TAG GGT TC | qPCR |
| *mas* F  *mas* R | CAT CAG GTG CAT AAC GTT GC  TCT GCT CAA AGG TGA TGT CG | qPCR |
