# Supplementary material for: Genome analysis identifies a spontaneous nonsense mutation in ppsD leading to attenuation of virulence in laboratory-manipulated Mycobacterium tuberculosis
Source: BMC Genomics. 2019 Feb 12;20:129. doi: 10.1186/s12864-019-5482-y (PMC6373159; doi:10.1186/s12864-019-5482-y)
Supplement: Supplementary file 2 — Table S2. List of SNPs identified M.tb Comp1 strain with respect to M.tb H37Rv. (DOCX 42 kb) [file 12864_2019_5482_MOESM2_ESM.docx]

**Table S2:** **List of SNPs identified *M.tb* Comp1 strain with respect to *M.tb* H37Rv**

| **SNP Location (H37Rv)** | **Rv Number** | **Gene Name** | **Nucleotide Change** | **Amino acid change** | **Type** | **Ioerger et al., 2010^1^** | **Cherny-aeva et al.,2014^2^** |
| --- | --- | --- | --- | --- | --- | --- | --- |
| 14785 | Rv0012 |  | T/C | C233R | N | Yes | Yes |
| 55553 | Rv0050 | ponA1 | C/T | P631S | N | Yes | Yes |
| 69989 | Rv0064 |  | G/A | G457D | N | Yes | Yes |
| 87163 | Intergenic |  | G/T | NA | NA | No | Yes |
| 90144 | Rv0082 |  | A/G | Q74R | N | Yes | No |
| 91071 | Rv0083 |  | T/C | I224I | S | Yes | No |
| 116000 | Rv0101 | nrp | T/G | V2000V | S | Yes | Yes |
| 131450 | Rv0109 | PE_PGRS1 | G/C | S23S | S | No | Yes |
| 132417 | Rv0109 | PE_PGRS1 | C/G | R346G | N | No | Yes |
| 161047 | Rv0133 |  | G/A | G60D | N | No | No |
| 213798 | Rv0182c | sigG | C/G | A115P | N | No | Yes |
| 234477 | Rv0197 |  | T/G | Y749STOP | N | Yes | Yes |
| 242299 | Rv0204c |  | C/G | V306L | N | Yes | No |
| 250575 | Rv0210 |  | C/G | G152G | S | No | Yes |
| 333892 | Rv0278c | PE_PGRS3 | G/C | R807G | N | No | Yes |
| 336681 | Rv0279c | PE_PGRS4 | C/G | P798R | N | No | Yes |
| 336682 | Rv0279c | PE_PGRS4 | C/G | P798A | N | No | Yes |
| 336689 | Rv0279c | PE_PGRS4 | C/G | S796R | N | No | Yes |
| 336694 | Rv0279c | PE_PGRS4 | T/G | S794A | N | No | Yes |
| 336698 | Rv0279c | PE_PGRS4 | G/C | G793G | S | No | Yes |
| 336701 | Rv0279c | PE_PGRS4 | T/C | G792G | S | No | Yes |
| 336707 | Rv0279c | PE_PGRS4 | C/T | D790D | S | No | Yes |
| 336708 | Rv3008 |  | A/G | S148S | S | No | Yes |
| 336710 | Rv0279c | PE_PGRS4 | T/C | A789A | S | No | Yes |
| 337959 | Rv0279c | PE_PGRS4 | A/C | I372S | N | No | Yes |
| 338020 | Rv0279c | PE_PGRS4 | A/C | C352G | N | No | Yes |
| 338100 | Rv0279c | PE_PGRS4 | T/C | N325S | N | No | Yes |
| 338453 | Rv0279c | PE_PGRS4 | A/G | A208A | S | No | Yes |
| 373570 | Rv3343c | PPE54 | C/A | G410G | S | No | Yes |
| 390828 | Rv0323c |  | T/C | S142G | N | Yes | Yes |
| 426909 | Rv0355c | PPE8 | A/C | W2591G | N | Yes | No |
| 458282 | Rv0382c | pyrE | A/G | Y33Y | S | Yes | No |
| 459399 | Intergenic |  | A/C | NA | NA | No | Yes |
| 467516 | Rv0388c | PPE9 | C/G | S163S | S | No | Yes |
| 467526 | Rv0388c | PPE9 | C/G | G159A | N | No | Yes |
| 467546 | Rv0388c | PPE9 | G/C | D153E | N | No | Yes |
| 467557 | Rv0388c | PPE9 | A/C | L149V | N | No | Yes |
| 467564 | Rv0388c | PPE9 | A/C | H147Q | N | No | Yes |
| 467585 | Rv0388c | PPE9 | G/C | H140Q | N | No | Yes |
| 467590 | Rv0388c | PPE9 | T/C | T138A | N | No | Yes |
| 467621 | Rv0388c | PPE9 | T/G | G128G | S | No | Yes |
| 467638 | Rv0388c | PPE9 | G/T | Q122K | N | No | Yes |
| 511518 | Rv0425c | ctpH | T/G | I1268I | S | Yes | No |
| 532097 | Rv0442c | PPE10 | T/C | K40E | N | Yes | No |
| 552085 | Rv0461 |  | A/G | Q20Q | S | Yes | No |
| 563577 | Rv0473 |  | A/G | K5R | N | Yes | No |
| 591170 | Rv0500A |  | C/A | A20A | S | No | Yes |
| 608331 | Rv0516c |  | T/G | M69L | N | No | No |
| 623472 | Rv0532 | PE_PGRS6 | A/G | D227G | N | No | Yes |
| 623508 | Rv0532 | PE_PGRS6 | C/G | A239G | N | No | Yes |
| 627025 | Rv0535 | pnp | G/C | R190P | N | No | Yes |
| 635633 | Rv0543c |  | C/T | A81A | S | No | No |
| 670530 | Rv0576 |  | T/G | V228G | N | No | Yes |
| 672491 | Rv0578c | PE_PGRS7 | C/G | G1143G | S | No | Yes |
| 781922 | Rv0682 | rpsL | A/G | K121K | S | Yes | No |
| 783558 | Rv0684 | fusA1 | C/G | G358G | S | No | Yes |
| 836272 | Rv0746 | PE_PGRS9 | A/G | E191G | N | No | Yes |
| 836291 | Rv0746 | PE_PGRS9 | A/G | G197G | S | No | Yes |
| 836426 | Rv0746 | PE_PGRS9 | A/C | L242L | S | No | Yes |
| 836454 | Rv0746 | PE_PGRS9 | A/G | T252A | N | No | Yes |
| 836538 | Rv0746 | PE_PGRS9 | A/G | N280D | N | No | Yes |
| 836658 | Rv0746 | PE_PGRS9 | A/G | T320A | N | No | Yes |
| 837033 | Rv0746 | PE_PGRS9 | A/G | T445A | N | No | Yes |
| 838990 | Rv0747 | PE_PGRS10 | C/G | A180A | S | No | Yes |
| 839123 | Rv0747 | PE_PGRS10 | A/G | R225G | N | No | Yes |
| 839129 | Rv0747 | PE_PGRS10 | C/G | R227G | N | No | Yes |
| 839194 | Rv0747 | PE_PGRS10 | A/G | T248T | S | No | Yes |
| 839334 | Rv0747 | PE_PGRS10 | A/G | K295R | N | No | Yes |
| 839348 | Rv0747 | PE_PGRS10 | A/G | S300G | N | No | Yes |
| 840496 | Rv0747 | PE_PGRS10 | C/G | G682G | S | No | Yes |
| 927110 | Rv0833 | PE_PGRS13 | A/G | S584G | N | No | Yes |
| 958922 | Rv0861c | ercc3 | C/A | A410A | S | No | No |
| 990001 | Rv0890c |  | G/C | P866A | N | Yes | Yes |
| 1000202 | Rv0896 | gltA2 | T/G | V244G | N | No | Yes |
| 1025106 | Rv0919 |  | T/C | F141F | S | Yes | Yes |
| 1037911 | Rv0930 | pstA1 | C/T | R305STOP | N | Yes | Yes |
| 1093406 | Rv0978c | PE_PGRS17 | A/G | V318V | S | No | Yes |
| 1175022 | Intergenic |  | G/C | NA | NA | No | Yes |
| 1175947 | Rv1052 |  | C/A | D75E | N | No | Yes |
| 1238725 | Rv1112 |  | C/A | R157R | S | No | Yes |
| 1244700 | Rv1121 | zwf1 | T/C | L332L | S | Yes | No |
| 1270900 | Rv1143 | mcr | C/G | A280G | N | No | Yes |
| 1313338 | Intergenic | NA | A/G | NA | NA | Yes | Yes |
| 1315191 | Rv1180 | pks3 | A/C | STOP489Y | N | Yes | Yes |
| 1315884 | Rv1181 | pks4 | G/A | A217A | S | Yes | Yes |
| 1327402 | Rv1185c | fadD21 | T/C | E37E | S | Yes | No |
| 1331696 | Rv1188 |  | A/C | R226R | S | Yes | No |
| 1414021 | Rv1266c | pknH | C/T | R607Q | N | Yes | Yes |
| 1453608 | Rv1297 | rho | T/C | G135G | S | Yes | No |
| 1471659 | Intergenic |  | C/T | NA | NA | Yes | Yes |
| 1500630 | Intergenic |  | G/C | NA | NA | No | Yes |
| 1555592 | Rv1382 |  | C/G | A39G | N | No | Yes |
| 1636991 | Rv1452c | PE_PGRS28 | T/C | G414G | S | No | Yes |
| 1685301 | Rv1493 | mutB | G/C | A433P | N | No | Yes |
| 1711627 | Rv1520 |  | C/T | Y200Y | S | No | No |
| 1901816 | Rv1677 | dsbF | A/G | Q23Q | S | Yes | No |
| 1925555 | Rv1699 | pyrG | G/C | G576A | N | No | Yes |
| 2006032 | Rv1771 |  | A/G | Q291R | N | No | No |
| 2020563 | Rv1783 |  | A/T | STOP463L | N | Yes | No |
| 2050913 | Rv1808 | PPE32 | A/G | E331E | S | Yes | No |
| 2051746 | Rv1809 | PPE33 | T/C | A155A | N | Yes | Yes |
| 2057774 | Rv1815 |  | A/T | I83F | N | Yes | Yes |
| 2070389 | Rv1823 |  | C/A | Q230K | N | No | Yes |
| 2082756 | Rv1836c |  | G/T | A628A | S | No | Yes |
| 2147399 | Rv1900c | lipJ | C/G | A79P | N | No | Yes |
| 2153410 | Rv1907c |  | A/G | V158A | N | No | Yes |
| 2166442 | Rv1917c | PPE34 | C/A | L291F | N | No | Yes |
| 2167489 | Intergenic |  | T/C | NA | NA | Yes | No |
| 2177654 | Rv1925 | fadD31 | A/C | M190L | N | Yes | No |
| 2221796 | Rv1979c |  | C/T | V457I | N | Yes | No |
| 2251999 | Intergenic | NA | A/G | NA | NA | Yes | Yes |
| 2282787 | Rv2037c |  | C/T | C312Y | N | Yes | Yes |
| 2297976 | Rv2048c | pks12 | G/A | S3004L | N | Yes | No |
| 2361623 | Rv2101 | helZ | A/C | M462L | N | Yes | No |
| 2387733 | Rv2126c | PE_PGRS37 | T/C | E81E | S | No | Yes |
| 2432178 | Intergenic |  | G/T | NA | NA | No | Yes |
| 2470149 | Rv2205c |  | T/C | E105E | N | Yes | No |
| 2505919 | Intergenic | NA | A/G | NA | NA | Yes | No |
| 2607657 | Rv2333c | stp | G/C | T222R | N | No | Yes |
| 2607658 | Rv2333c | stp | T/C | T222A | N | No | Yes |
| 2618405 | Rv2340c | PE_PGRS39 | G/C | G169G | S | No | Yes |
| 2632918 | Intergenic |  | G/C | NA | NA | No | Yes |
| 2632922 | Intergenic |  | G/C | NA | NA | No | Yes |
| 2718852 | Intergenic | none | T/G | NA | NA | Yes | Yes |
| 2748136 | Rv2448c | valS | G/C | A697G | N | No | Yes |
| 2751804 | Rv2450c | rpfE | C/T | R126Q | N | Yes | Yes |
| 2781911 | Rv2476c | gdh | C/A | G118Stop | N | No | Yes |
| 2809621 | Rv2495c | pdhC | T/C | T107A | N | Yes | Yes |
| 2844879 | Rv2524c | fas | G/C | A1485G | N | No | Yes |
| 2850341 | Rv2525c |  | G/T | G79G | S | No | Yes |
| 2943411 | Rv2614A |  | T/C | L12L | S | Yes | No |
| 2954439 | Rv2627c |  | T/C | R104G | N | Yes | Yes |
| 2996194 | Rv2680 |  | T/A | V30V | S | Yes | No |
| 3012293 | Rv2695 |  | A/G | T126T | S | Yes | No |
| 3054724 | Rv2741 | PE_PGRS47 | A/G | S271G | N | No | Yes |
| 3091658 | Rv2783c | gpsI | C/G | A314P | N | No | Yes |
| 3205978 | Rv2896c |  | A/C | S153A | N | Yes | No |
| 3248308 | Rv2931 | ppsA | A/C | H955P | N | No | Yes |
| 3254365 | Rv2932 | ppsB | T/C | L1098L | S | Yes | No |
| 3267020 | Rv2934 | ppsD | G/A | W1591Stop | N | No | No |
| 3370177 | Rv3011c | gatA | T/G | M420L | N | Yes | No |
| 3379751 | Intergenic |  | A/C | NA | NA | No | Yes |
| 3379757 | Intergenic |  | A/C | NA | NA | No | Yes |
| 3379763 | Intergenic |  | G/A | NA | NA | No | Yes |
| 3379784 | Intergenic |  | C/A | NA | NA | No | Yes |
| 3379788 | Intergenic |  | C/G | NA | NA | No | Yes |
| 3510642 | Rv3144c | PPE52 | T/C | S226G | N | Yes | No |
| 3534314 | Rv3165c |  | C/G | V28L | N | No | Yes |
| 3547816 | Rv3179 |  | C/G | R67G | N | No | Yes |
| 3718357 | Rv3331 | sugI | C/T | P423L | N | Yes | Yes |
| 3759060 | Rv3350c | PPE56 | C/A | L2682F | N | No | Yes |
| 3818332 | Rv3401 |  | C/G | D97E | N | No | Yes |
| 3862473 | Intergenic |  | A/C | NA | NA | No | Yes |
| 3896340 | Rv3479 |  | T/G | L174R | N | Yes | Yes |
| 3934699 | Rv3508 | PE_PGRS54 | G/A | S1232N | N | No | Yes |
| 3934733 | Rv3508 | PE_PGRS54 | G/C | G1243G | S | No | Yes |
| 3934734 | Rv3508 | PE_PGRS54 | G/A | A1244T | N | No | Yes |
| 3940802 | Rv3511 | PE_PGRS55 | A/G | N396D | N | No | Yes |
| 3948347 | Rv3514 | PE_PGRS57 | T/G | S852A | N | No | Yes |
| 3948355 | Rv3514 | PE_PGRS57 | T/G | G854G | S | No | Yes |
| 3948356 | Rv3514 | PE_PGRS57 | A/G | S855G | N | No | No |
| 3948359 | Rv3514 | PE_PGRS57 | A/C | S856G | N | No | Yes |
| 3948404 | Rv3514 | PE_PGRS57 | T/G | C871G | N | No | Yes |
| 3948414 | Rv3514 | PE_PGRS57 | T/G | V874G | N | No | Yes |
| 3948417 | Rv3514 | PE_PGRS57 | T/G | V875G | N | No | Yes |
| 3949000 | Rv3514 | PE_PGRS57 | G/C | G1069G | S | No | Yes |
| 3949001 | Rv3514 | PE_PGRS57 | G/A | A1070T | N | No | Yes |
| 3973911 | Rv3535c | hsaG | T/G | D197A | N | No | Yes |
| 4147070 | Rv3704c | gshA | A/G | L373L | S | Yes | No |
| 4169712 | Rv3724B | cut5b | A/C | Y36S | N | No | Yes |
| 4407904 | Rv3919c | gidB | G/A | S100F | N | Yes | No |

SNPs which are unique to Comp1 strain are colored in blue.

S : Synonymous

N : Non-Synonymous

NA : SNP falling into intergenic region

^1^Ioerger, T.R., et al., *Variation among genome sequences of H37Rv strains of Mycobacterium tuberculosis from multiple laboratories.* J Bacteriol, 2010. **192**(14): p. 3645-53.

^2^Chernyaeva, E.N., et al., *Genome-wide Mycobacterium tuberculosis variation (GMTV) database: a new tool for integrating sequence variations and epidemiology.* BMC Genomics, 2014. **15**: p. 308.
