# Supplementary material for: Genome analysis identifies a spontaneous nonsense mutation in ppsD leading to attenuation of virulence in laboratory-manipulated Mycobacterium tuberculosis
Source: BMC Genomics. 2019 Feb 12;20:129. doi: 10.1186/s12864-019-5482-y (PMC6373159; doi:10.1186/s12864-019-5482-y)
Supplement: Supplementary file 3 — Table S3. Insertions and Deletions (InDels) identified in M. tb Comp1 strain with respect to M. tb H37Rv. (DOCX 20 kb) [file 12864_2019_5482_MOESM3_ESM.docx]

**Table S3:** **Insertions and Deletions (InDels) identified in *M. tb* Comp1 strain with respect to *M. tb* H37Rv**

| **InDel Position (H37Rv)** | **Rv number** | **Gene Name** | **Ref Nucleotide (H37Rv)** | **Alt Nucleotide (comp1)** | **Indel*** | **nucleotide changed number** | **Ioerger et al., 2010^1^** | **Cherny-aeva et al.,2014^2^** |
| --- | --- | --- | --- | --- | --- | --- | --- | --- |
| 55533 | Rv0050 | ponA1 | TGCCGCCGCCGCCGCCGCCGCCG | TGCCGCCGCCGCCGCCGCCG | -GCC | -3 | No | Yes |
| 131174 | Intergenic | NA | TGG | TGGG | +G | 1 | Yes | Yes |
| 234496 | Rv0197 |  | C | CGT | +GT | 2 | Yes | Yes |
| 424320 | Rv0354c | PPE7 | GGA | GGGA | +C | 1 | Yes | Yes |
| 467508 | Rv0388c | PPE9 | CG | CCG | +G | 1 | No | Yes |
| 976897 | Rv0878c | PPE13 | A | CCCAA | +G | 4 | No | Yes |
| 1010204 | Rv0907 |  | CGG | CGGG | +G | 1 | Yes | Yes |
| 1168715 | Rv1046c |  | AAG | AAAG | +T | 1 | Yes | Yes |
| 1313337 | Intergenic | NA | A | AG | +G | 1 | Yes | Yes |
| 1780586 | Rv1575 |  | CG | CGG | +G | 1 | Yes | Yes |
| 1987087 | Rv1755c | plcD | C | TTC | +AA | 2 | No | Yes |
| 2207591 | Intergenic | NA | T | TC | +C | 1 | Yes | Yes |
| 2523205 | Intergenic | NA | GCG | GCGCCG | +CGC | 3 | Yes | Yes |
| 2525722 | Rv2250A |  | CGGGGG | CGGGG | -G | -1 | Yes | Yes |
| 3415180 | Intergenic | NA | ACACCTAGGGGGTGGCACCTAGGGGGTGGCAC | ACACCTAGGGGGTGGCAC | -CACCTAGGGGGTGG | -14 | No | Yes |
| 3580637 | Intergenic | NA | TTT | TT | -T | -1 | Yes | Yes |
| 3590686 | Intergenic | NA | GT | GCT | +C | 1 | Yes | Yes |
| 3779671 | PE_PGRS51 | Rv3367 | CGGCAACGGTGGCAACGGTGGCAACGGTG | CGGCAACGGTGGCAACGGTGGCAACGGTGGCAACGGTG | +GGCAACGGT | 9 | No | Yes |
| 3862472 | Intergenic | NA | GAA | GA | -A | -1 | Yes | Yes |
| 4095002 | Rv3655c |  | GTC | TC | -G | -1 | Yes | Yes |
| 4400660 | Rv3911 | sigM | ACCC | ACC | -C | -1 | Yes | Yes |

*+ denotes insertion/ - denotes deletion

NA : SNP falling into intergenic region

^1^Ioerger, T.R., et al., *Variation among genome sequences of H37Rv strains of Mycobacterium tuberculosis from multiple laboratories.* J Bacteriol, 2010. **192**(14): p. 3645-53.

^2^Chernyaeva, E.N., et al., *Genome-wide Mycobacterium tuberculosis variation (GMTV) database: a new tool for integrating sequence variations and epidemiology.* BMC Genomics, 2014. **15**: p. 308.

**Table S4:** **Total counts per minute (CPM) obtained on incorporation of ^14^C-propionate into *M. tb* cultures**

| **Sample** | **Experiment 1** | | **Experiment 2** | | ***p*value*** |
| --- | --- | --- | --- | --- | --- |
|  | **CPM for 5 ml culture** | **Fold difference** | **CPM for 5 ml culture** | **Fold difference** |  |
| H37Rv | 601900 | 92.6 | 582850 | 153.38 | 0.0003 |
| Mut1 | 1110100 | 170.78 | 919400 | 241.95 | 0.0088 |
| Comp1 | 6500 | 1 | 3800 | 1 | - |
| Comp9 | 400400 | 61.6 | 389900 | 102.6 | 0.0002 |
| Comp11 | 658900 | 101.37 | 618950 | 162.88 | 0.0010 |

The counts from two separate experiments are shown in the table

*pvalue is calculated for the difference in count for each strain vs. Comp1.
