# Supplementary material for: Genome analysis identifies a spontaneous nonsense mutation in ppsD leading to attenuation of virulence in laboratory-manipulated Mycobacterium tuberculosis
Source: BMC Genomics. 2019 Feb 12;20:129. doi: 10.1186/s12864-019-5482-y (PMC6373159; doi:10.1186/s12864-019-5482-y)
Supplement: Supplementary file 4 — Table S4. Total counts per minute (cpm) obtained on incorporation of 14C-propionate into M. tb cultures. (DOCX 14 kb) [file 12864_2019_5482_MOESM4_ESM.docx]

**Table S4:** **Total counts per minute (cpm) obtained on incorporation of ^14^C-propionate into *M. tb* cultures**

| **Strain** | **Average cpm for 5 ml culture** | **Relative incorporation efficiency of ^14^C-propionate (%)** |
| --- | --- | --- |
| H37Rv | 592375 | 100 |
| Mut1 | 1014750 | 171.3 |
| Comp1 | 5150 | 0.9 |
| Comp9 | 395150 | 66.7 |
| Comp11 | 638925 | 107.9 |

Individual cpm values of two separate experiments did not vary more than 10% of the average cpm.
